# In Vitro Toxicity Assessment of Tire-related Chemicals in Cells from Diverse Fish Species

**DOI:** 10.1007/s00128-026-04314-y

**Published:** 2026-08-11

**Authors:** Akihiro Moriyama, Masanori Horie, Wataru Naito

**Affiliations:** 1 National Institute of Advanced Industrial Science and Technology (AIST), Research Institute of Science for Safety and Sustainability, Tsukuba, Ibaraki 305-8569 Japan; 2 National Institute of Advanced Industrial Science and Technology (AIST), Health and Medical Research Institute, Takamatsu, Kagawa 761-0395 Japan; 3 National Institute of Advanced Industrial Science and Technology (AIST), Integrated Research Center for Nature Positive Technology, Tsukuba, Ibaraki 305-8569 Japan

**Keywords:** 6PPD-quinone, Cytotoxicity, Tire road wear particles, Salmon, In vitro assay

## Abstract

Urban stormwater runoff introduces tire‑related contaminants such as 6PPD‑quinone (6PPD‑Q), which has been shown to exhibit acute toxicity in some salmonid species, although the underlying toxic mechanisms remain incompletely understood. To expand in vitro toxicological assessments, we evaluated the cytotoxicity of 3,4‑dichloroaniline and 6PPD‑quinone using six fish‑derived cell lines from salmonids and phylogenetically distant species, based on nominal concentrations at the time of addition. Among these cell lines, CSE‑119 cells, which are derived from coho salmon, exhibited the highest sensitivity to 6PPD‑quinone (EC_50_ = 8.54 µg/L), demonstrating pronounced species‑specific differences in sensitivity to quinone‑type toxicants and underscoring the value of comparative in vitro models. Using multiple fish cell lines enables the efficient, animal‑welfare–conscious screening of tire‑associated chemicals. However, further investigation is needed into evaluation methods that take into account factors such as the stability of chemicals in the culture medium.

## Introduction

6PPD‑quinone (6PPD‑Q), an oxidation product of the widely used rubber antioxidant N‑(1,3‑dimethylbutyl)‑N′‑phenyl‑p‑phenylenediamine (6PPD), was identified as the cause of mass mortality in coho salmon (*Oncorhynchus kisutch*) returning to urban streams (Tian et al. 2021). This finding underscored the need to better understand the toxicity and species‑specific sensitivity of 6PPD‑Q.

In this context, in vitro assays using fish‑derived cell lines have emerged as a promising alternative to whole‑organism testing, enabling efficient toxicity screening while reducing animal use. Previous studies have demonstrated that fish cell‑based assays can reproduce known species sensitivities to 6PPD‑Q, particularly in coho salmon‑derived cell lines (Greer et al. 2023; Mano et al. 2025; Nair et al. 2025). Mechanistic investigations have further suggested that mitochondrial dysfunction and interspecies differences in metabolic processing may contribute to variable sensitivity among fish species (Mahoney et al. 2022; Montgomery et al. 2023). However, available in vitro cell‑based studies have largely been restricted to salmonids, and cytotoxicity data across broader phylogenetic groups of fish remain limited.

Here, we assessed the cytotoxicity of 6PPD-Q in six fish cell lines from phylogenetically distinct species after validating assay performance with the reference toxicant 3,4-dichloroaniline (3,4-DCA). The findings of this study lend support the utility of fish cell-based assays for evaluating species-specific sensitivity to tire-related chemicals. However, it should be noted that all exposure concentrations were nominal values and were not analytically confirmed. Therefore, further studies addressing chemical stability and actual exposure concentrations are needed.

## Materials and Methods

### Chemicals and Preparation of Sample Solution

All the chemicals used for cytotoxicity experiments were purchased from FUJIFILM Wako Pure Chemical Corporation (Osaka, Japan) unless stated otherwise. 6PPD-Q was purchased from Toronto Research Chemical (Toronto, Ontario, Canada). The test chemicals were dissolved in dimethyl sulfoxide (DMSO). The concentration ranges for 3,4-DCA and 6PPD-Q were selected based on previously reported toxicity values, including the OECD TG249 reference EC_50_ (50% effective concentration) value of 43.6 ± 6.1 mg/Lfor 3,4-DCA (OECD 2021) and the EC_50_ value of 7.8 µg/L for 6PPD-Q in CSE-119 cells (Greer et al. 2023). Stock solutions of 3,4-DCA (80 mg/mL) and 6PPD-Q (1 mg/mL) were stored at − 20 °C and diluted 400-fold and sequentially 100-fold and 20-fold, respectively, to achieve maximum test concentrations of 200 mg/L and 500 µg/L.

### Cell Culture

Fish cell lines derived from four salmonid species and two phylogenetically distant fish species were used here. Specifically, the salmonid cell lines included RTG-2 (ECACC 90102529; Rainbow trout, *Oncorhynchus mykiss*), CSE-119 (ECACC 95122019; Coho salmon, *O*. *kisutch*), CHSE-214 (ECACC 91041114; Chinook salmon, *O*. *tshawytscha*), and SSE-5 (ECACC 95122021; sockeye salmon, *O*. *nerka*). The non-salmonid fish cell lines were BF-2 (ECACC 00021712; Bluegill, *Lepomis macrochirus*) and FHM (ECACC 88102401; Fathead minnow, *Pimephales promelas*). All cell lines were purchased from UK Health Security Agency (Porton Down, Salisbury, United Kingdom) via KAC, Co., Inc. (Kyoto, Japan). RTG-2, CSE-119, CHSE-214, and SSE-5 cell lines were incubated at 19 °C, while BF-2 and FHM cell lines were incubated at 26 °C. All cell lines were routinely cultured in L-15 medium (Leibovitz’s L-15, FUJIFILM Wako Pure Chemical Corporation) supplemented with 10% fetal bovine serum (FBS), 100 U/mL penicillin, and 100 µg/mL streptomycin.

### Cell Viability Assay

Cell viability was assessed using the Alamar Blue assay (Invitrogen; Thermo Fisher Scientific Inc., Waltham, Massachusetts, USA) based on cellular metabolic activity as previously described (Mano et al. 2025). Briefly, cells were seeded in black-bottom 96-well plates (20000 cells/well; SSE-5: 10000 cells/well) and allowed to attach for 24 h. Cells were then exposed to test compounds in FBS-free L-15 medium for 24 h using two-fold serial dilutions (3,4-DCA, starting at 200 mg/L; 6PPD-Q, starting at 500 µg/L). Each plate contained 4 replicates per concentration per cell (*n* = 4). After exposure, cells were washed with HBSS and incubated with 10% (v/v) Alamar Blue solution. Fluorescence was measured at 535/590 nm (Tecan Infinite 200 Pro, Tecan, Männedorf, Switzerland), and cell viability was normalized to solvent controls.

### Calculations of Cytotoxicity Values

Cell viability in cytotoxicity tests was statistically analyzed using R (version 4.2.2) to determine the EC_50_ at 24 h, along with the corresponding 95% confidence intervals, using the drc package. For the cytotoxicity data, a four-parameter log-logistic (LL.4) model with a Gaussian error distribution was fitted using the drm function.

## Results and discussion

### 3,4-DCA Cytotoxicity Across Multiple Fish Cell Lines

**Fig. 1:**
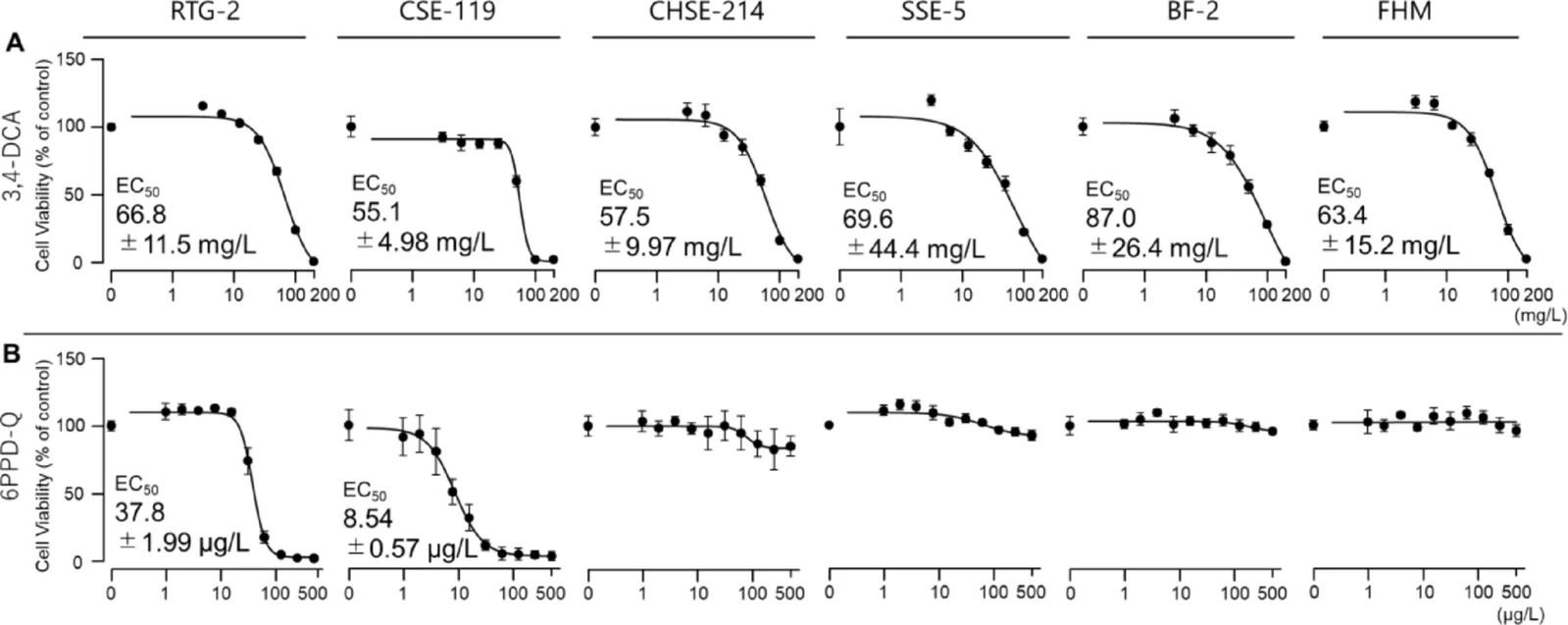
Metabolic effects of 3,4-dichloroaniline (3,4-DCA, **A**) and 6PPD-Q (**B**) in fish cell lines. Metabolic activity 24 h after exposure to a concentration gradient of 3,4-DCA (3.125–200 mg/L) and 6PPD-quinone (6PPD-Q; 0.98–500 µg/L). Each concentration was tested in 4 replicates

Here, we examined the cytotoxicity of 3,4-DCA in six fish-derived cell lines after 24 h of exposure using the Alamar Blue assay. 3,4-DCA caused a concentration-dependent decrease in cell viability across all cell lines, with EC_50_ value of ~ 50 mg/L (Fig. 1A). This value was close to that of the previously reported EC_50_ (43.6 ± 6.1 mg/L) for RTgill-W1 cells under OECD Test Guideline 249 (TG 249; 2021). TG 249 is a standardized in vitro assay using the RTgill-W1 cell line to assess acute chemical toxicity by measuring cellular activity after 24 h of exposure. Therefore, the consistency between our results and those obtained using TG 249 assay validates the technical reliability of our protocol. Furthermore, no marked differences in sensitivity were observed among the salmonid-derived cell lines, and CSE-119 cells were not particularly sensitive compared with the other tested cell lines.

### Cytotoxicity of 6PPD-Q to Fish Cells

Among the tested fish-derived cell lines, CSE-119 cells exhibited the highest sensitivity to 6PPD-Q (EC_50_ = 8.54 µg/L), followed by RTG-2 cells (EC_50_ = 37.8 µg/L) (Fig. 1B). No significant cytotoxicity was observed in CHSE-214, SSE-5, BF-2, or FHM cells. The EC_50_ value for CSE-119 cells was consistent with that reported by Greer et al. (2023) (7.8 µg/L), further supporting the utility of this cell line as an in vitro model for evaluating 6PPD-Q toxicity.

In contrast, a discrepancy was observed for RTG-2 cells. Although Greer et al. (2023) reported no significant metabolic effects at concentrations up to 100 µg/L, we detected a measurable cytotoxic response, with an EC_50_ of 37.8 µg/L. Considering that both studies employed the Alamar Blue assay, this variation may be attributable to differences in experimental details such as cell condition, culture parameters (serum supplementation, incubation time), or subtle procedural variations. Collectively, these results highlight the importance of standardized protocols when comparing cytotoxicity data across laboratories.

Solhaug et al. (2023) demonstrated that fish gill epithelial cell lines derived from different salmonid species exhibit distinct responses to oxidative stress, possibly due to differences in mitochondrial status and antioxidant defenses such as glutathione and catalase activities. Therefore, the interspecies variability in sensitivity may be partly attributed to differences in the expression or activity of oxidative stress-related enzymes among fish species. We primarily focused on evaluating cytotoxic responses and did not investigate the underlying mechanisms of toxicity. Although effects on mitochondria or other intracellular targets are plausible (Mahoney et al. 2022), elucidating the precise mechanisms of action—such as oxidative stress or mitochondrial dysfunction—requires further investigation. Additionally, the EC_50_ values reported here were calculated based on nominal exposure concentrations. Possible losses due to chemical adsorption to culture plates or pipette tips as well as degradation of 6PPD-Q including possible hydrolytic transformation (Di et al. 2022), were not quantified, which may have led to an underestimation of actual cellular exposure concentrations.

Conclusively, we corroborate earlier findings by confirming the high sensitivity of CSE-119 cells, derived from coho salmon, to 6PPD-Q exposure. In addition to salmonid cell lines, we evaluated the cytotoxicity of tire-related chemicals in several genetically distant fish cell lines. Collectively, these results highlight the utility of fish cell-based assays for screening tire-related chemicals and the importance of using multiple fish-derived cell lines. However, all exposure concentrations applied in this study were nominal and were not analytically confirmed, indicating that further studies incorporating exposure verification are needed. Additionally, as high-throughput cell-based assays are currently performed using different methodologies across research groups, an urgent exists need to establish harmonized protocols to ensure consistency and comparability of results. A limitation of this study is that the cell lines used differ in their origins (e.g., CSE-119 is embryonic, whereas FHM is epithelial). As metabolic activity varies among tissues, their sensitivities may also differ. Considering that fish primarily absorb chemicals through the gills, gill-derived cells may exhibit higher sensitivity (Mahoney et al. 2022). Future studies should evaluate such cell types to clarify sensitivity differences among tissues as well as between whole organisms and isolated cells. Furthermore, comparing sensitivities observed in fish cell lines with species-specific sensitivities reported in whole-organism toxicity tests will help clarify the ecological relevance and predictive value of in vitro assays for assessing 6PPD-Q toxicity.
